# ASAS-NANP SYMPOSIUM: mathematical modeling in animal nutrition—data-efficient computer vision for precision livestock farming

**DOI:** 10.1093/jas/skag316

**Published:** 2026-09-29

**Authors:** Yalong Pi, Karun Kaniyamattam, Adeolu Adekunle, Aniruddh Adiga, Luis O Tedeschi, Nick Duffield, Cheng Zhang

**Affiliations:** Institute of Data Science, Texas A&M University, College Station, TX 77843, United States; Department of Animal Science, Texas A&M University, College Station, TX 77843, United States; Department of Animal Science, Texas A&M University, College Station, TX 77843, United States; Institute of Data Science, Texas A&M University, College Station, TX 77843, United States; Department of Animal Science, Texas A&M University, College Station, TX 77843, United States; Department of Electrical and Computer Engineering & Institute of Data Science, Texas A&M University, College Station, TX 77843, United States; Department of Computer Science, Texas A&M University, College Station, TX 77843, United States

**Keywords:** data-efficient AI, computer vision, individual animal identification, artificial intelligence, precision livestock farming

## Abstract

Precision livestock farming (PLF) increasingly relies on computer vision (CV) to monitor animal behavior, health, and production. However, widespread adoption remains limited by the large quantities of manually labeled data required to train most artificial intelligence (AI) models. This paper reviews AI and CV applications in PLF and presents three data-efficient case studies conducted at a research feedlot at Texas A&M University. The assessment of AI model predictability was conducted with precision, recall, F1-score, and adjusted rand index (ARI). In Case Study 1, a data-efficient Mask R-CNN (region-based convolutional neural network) model pre-trained on the common objects in context (COCO) dataset was applied without modification to detect cattle feeding events at individual feed bunks. Across 98,720 frame-bunk classifications, the system achieved 87.2% precision, 89.1% recall, and an 88.2% F1 score compared to physical bunk sensor ground truth. In Case Study 2, transfer learning on a ResNet backbone was used to classify bunk feed grade across six customized levels (S00–S40) using only 183 labeled images. The model achieved 91% accuracy on an independent verification set of 7,905 images. In Case Study 3, statistical tools, i.e. Density-Based Spatial Clustering of Applications with Noise (DBSCAN) and k-means clustering, were applied to identify individual animals at the bunk without manual animal-level annotation. This method and achieved an ARI of 0.96 against expert-verified ground truth, successfully differentiating animals. Integrating all three components, the framework produced a continuous record of individual animal bunk visits and estimated feed intake from a 1-h video. Key remaining challenges include long-term individual animal re-identification under changing appearance and environmental conditions, incorporation of spatiotemporal information to better characterize feeding behavior, and the limited availability of large-scale open datasets for PLF research. Future research will integrate multispectral imaging and audio analytics to improve model robustness and advance individual animal identification for cattle health and nutritional management.

## Teaser

Camera-based computer vision can identify individual cattle, detect feeding behavior, and grade feed levels with little to no manual data labeling, making affordable, non-invasive livestock monitoring practical for commercial farms. This data-efficient approach opens a path toward earlier illness detection, more precise feed management, and reduced antibiotic use.

## Introduction

Increased incomes, population growth, urbanization across regions, and dietary changes in emerging countries have driven unprecedented global demand for livestock products (Godde et al. 2018). Precision Livestock Farming (PLF) systems, incorporating a wide range of sensors to access production-related information, can potentially aid efficient livestock management (Banhazi et al. 2012). The wave of artificial intelligence (AI) is impacting PLF by supplying cost-effective sensing and modeling tools to optimize farming management (Tedeschi et al. 2025). AI applications in livestock systems are rapidly expanding across health, reproduction, nutrition, and behavior monitoring domains (Condotta and Tedeschi 2026), with computer vision (CV) emerging as one of the most scalable and non-invasive modalities.

Current CV has experienced significant advancements driven by the increasing power of computers and neural network (NN) developments. While the concept of NN existed for a long time for mapping complex input-output pairs, it was not until the introduction of backpropagation that such networks could be practically optimized (Rumelhart et al. 1986) using the chain rule in calculus. With the introduction of large datasets such as ImageNet (Russakovsky et al. 2015) and parallel computing devices such as graphics processing units (GPU), deep NN (DNN) showed significant advancements in CV (Krizhevsky et al. 2012), and natural language processing (NLP) (Vaswani et al. 2017). Because the practitioners best positioned to adopt these tools often lack a technical background in AI or CV, some fundamental AI concepts and key technical details are presented in Supplementary Appendix I Figures S1 and S2 (see online supplementary material for a color version of these figures). This material is essential to understanding the non-linear approximating power of DNNs.

Many CV applications in PLF rely on on-site cameras to capture videos made of frames (images) at a specific rate, usually 30 frames per second (FPS) for real-time, but sometimes lower to save space, especially in monitoring systems. CV convolutional neural network (CNN) algorithms can extract meaningful features, including shape, color, patterns, and more, by utilizing DNN to learn the high-dimensional relationships between the digital input and the numerical output (LeCun et al. 2015). For instance, ResNet (He et al. 2016) for image classification, you only look once (YOLO) series (Redmon et al. 2016) and single shot detection (SSD) (Liu et al. 2016) for object detection, Mask RCNN (He et al. 2017), and SegNet (Badrinarayanan et al. 2017) for pixel segmentation, generative adversarial networks (GANs) (Goodfellow et al. 2014) and their variations for image generation.

Most AI applications in PLF use CV models as a non-invasive sensing method to extract animal-specific information, such as behavior, health indicators, body condition, and movement patterns (Guarnido-Lopez et al. 2024). Cameras, combined with CV algorithms, provide a sensing and analysis framework that can complement many existing PLF technologies (Tedeschi et al. 2021). The typical CV tasks include phenotyping, body measurements, production evaluation, and animal tracking and behavior analysis (Fernandes et al. 2020). Depending on the function designs, the CV models can classify, localize (with bounding boxes), and segment targets at the pixel level, which are applied across a range of PLF endpoints: food intake (Bresolin et al. 2023), daily weight gain (Cominotte et al. 2020), body weight prediction (Gjergji et al. 2020), body condition score (Qiao et al. 2021), respiratory rate (Wu et al. 2020), and metabolism detection (Novais et al. 2019). Each of these applications is task-specific and trained on manually annotated data particular to that task, underscoring the same labeling bottleneck this paper’s case studies aim to review.

More recent CV in PLF work spans a wide range of tasks and model types: 3D animal reconstruction from 2D images via diffusion models (Jakab et al. 2023), pose estimation using vision mamba architectures (Daadouch et al. 2025), feeding-behavior detection via object detection (Bresolin et al. 2023), behavior classification via recurrent networks (Guo et al. 2021), interactive platforms for motion tracking (De Castro et al. 2025), and activity indexing (Saeidifar et al. 2025). Despite this diversity of tasks and architectures, nearly all these approaches share a common dependency: they still require hundreds to thousands of manually labeled images or video frames. This gap motivates the present work, which reviews three tutorial-style case studies to showcase the rationale for the methods: an off-the-shelf model when no labels exist, transfer learning when a small, labeled dataset is available, and label-free statistical clustering with pre-trained models. The advantages, limitations, and performance will be reviewed with evidence.

## Case studies

### Case study 1: off-the-shelf Mask-RCNN application for feed time sensing

This study tries to use the off-the-shelf CV model and weights without modification as the starting point to sense feed events for PLF. It is possible to detect pen-fed beef cattle feeding behavior using Mask RCNN (He et al. 2017), which was pre-trained on the Common Objects in Context (COCO) (Lin et al. 2014) dataset. In total, the COCO dataset covers 80 different daily object classes, e.g. cars and people, and already includes the class “cow” with 2,055 instances (labeled cows). Even though the cows in the COCO dataset are slightly different from the animals in our experiment, this allows a feasibility test. Manually defined feed bunk polygons were used to compare with these segmentation masks to derive feeding time for each bunk. Unlike retraining the Mask RCNN model (Qiao et al. 2019), off-the-shelf pre-trained models are widely available and do not require manual labels.

Figure 1 shows the methodology of this study, where the input is represented by green blocks and the framework output by blue blocks. All the feed bunk polygons need to be manually defined by using free online tools, e.g. the VIA annotation tool (Dutta and Zisserman 2019). It is worth noting that this annotation should be done only once with fixed cameras. This task also requires the feeding bunk opening to be both visible and substantial in size. Following the steps in Figure 1, each video frame is processed by the Mask R-CNN model, which produces pixel masks. Next, we compare each mask with each feeding bunk polygon geometrically. If there is an overlap between a mask and a polygon, we classify this as an indication of feeding at this bunk at the frame time T. Lastly, the CV classification records are compared with the physical bunk sensor classification.

**Figure 1:**
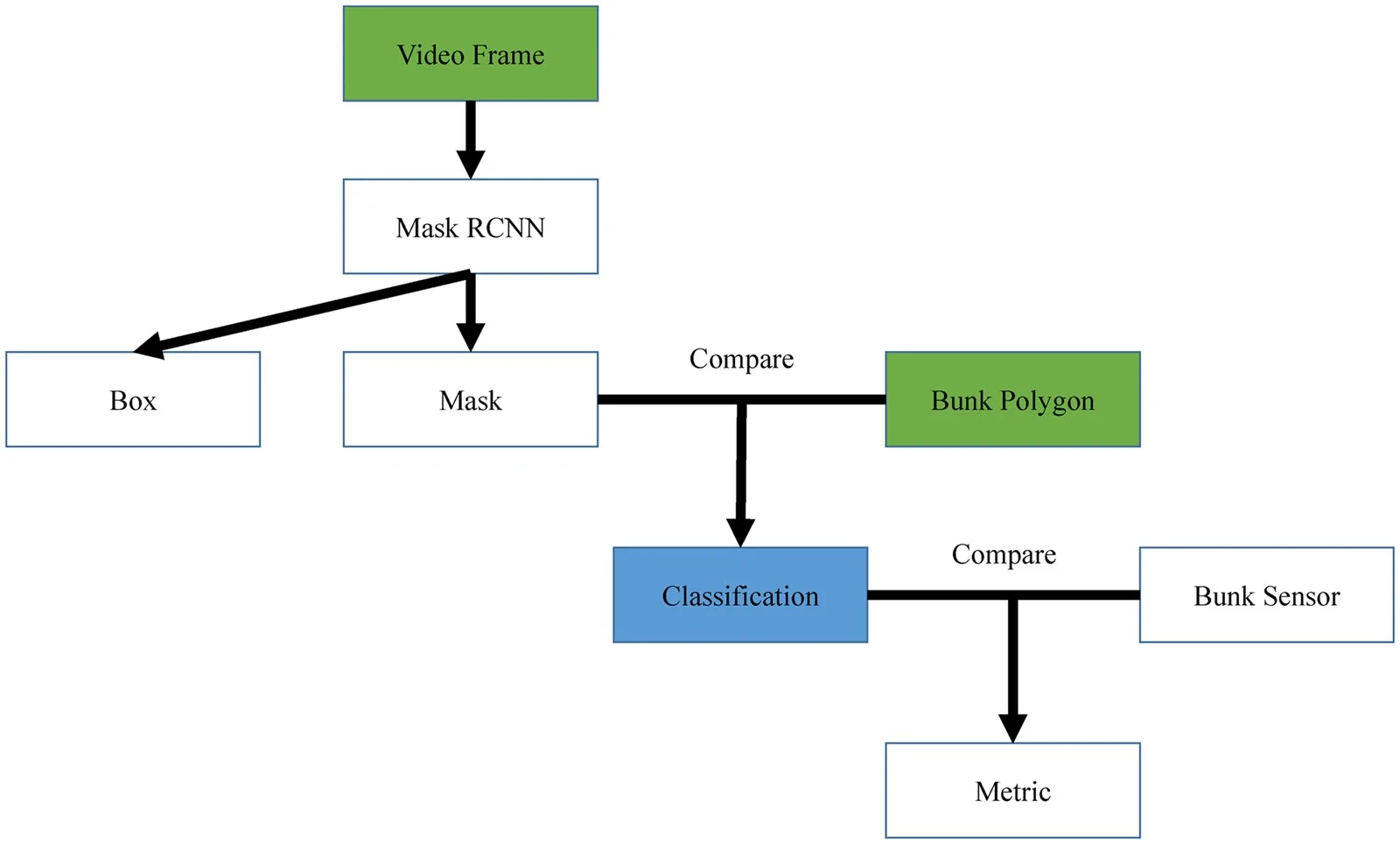
Case Study 1: off-the-shelf Mask RCNN application for feed time detection.

The experimental research feedlot station is located at the Beef Cattle Systems (BCS) from Texas A&M University, College Station, TX. Video data were recorded during the day by a camera (Microsoft LifeCam) at 1,280 × 720 resolution on June 19th of 2022. The feeding events were obtained by using GrowSafe sensors that record feed events with a start time, bunk, and duration, which were later converted to binary data at the video frame time level serving as the ground truth (GT). Figure 2 provides an example video frame of a pen with four feeding bunks numbered 13 to 16. Each feed bunk was manually marked with a yellow polygon. When the colored pixel mask overlaps with any yellow bunk polygons, such as the area filled with grid in Figure 2, the CV classification for that bunk is defined as feeding (1), and the corresponding GT data (1 or 0) will be used to benchmark its performance. For instance, one cattle feed at 7:36:15 AM at feed bunk 13. Because performance was assessed at the frame level, aligning the two independently operating recording systems’ clocks was essential to the study’s methodology. The GrowSafe bunk system and the LifeCam camera each timestamp their records using their own internal system clock. Both devices’ clocks were set to the same wall-clock time (the current date and time of day) for synchronization.

**Figure 2:**
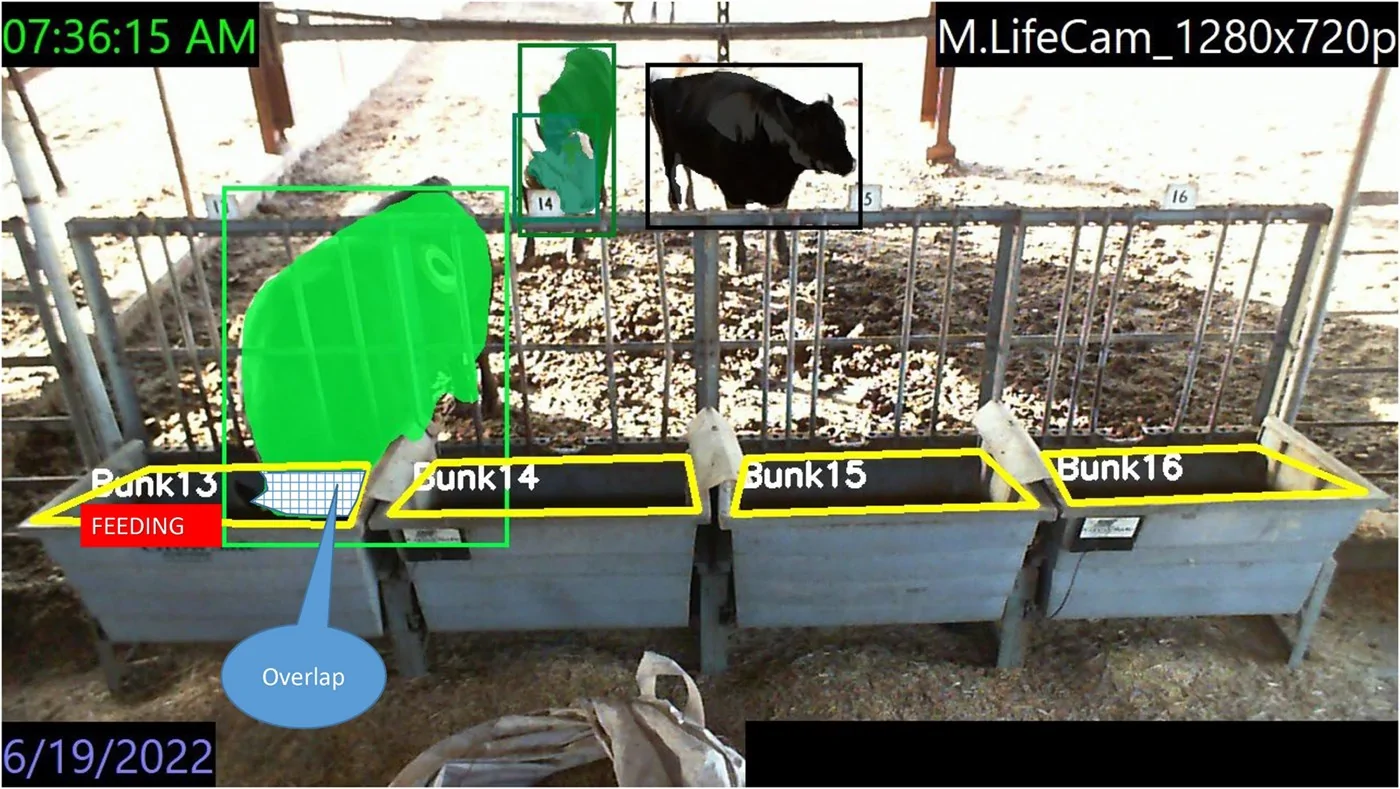
Example of computer vision feeding sensing.

#### Prediction evaluation

Based on the camera frame rate (10 FPS) and the video’s starting time, we calculated the corresponding time T for each frame. For each bunk at time T, we compared the CV and bunk sensor measurements GT. For instance, a feeding is sensed for 10 s, given the FPS = 10, a total of T1 to T100 are marked as feeding (1) for GT. By matching the CV and GT at the frame level, each comparison is determined as true positives (TP), false positives (FP), false negatives (FN), and true negatives (TN) cases. A TP case occurs when a bunk in a frame is predicted as feeding which aligns with the GT classification of feeding. On the other hand, an FP case arises when the CV model mistakenly identifies a bunk in a frame as feeding, despite GT indicating no feeding. TN represents cases where both CV and GT classify a bunk in a frame as not feeding, while an FN occurs when the CV suggests a bunk in a frame is not feeding, but the GT indicates feeding. The following precision, recall, and F1 score calculations are in Supplementary Appendix I.

#### Performance

A total of 98,720 cases is classified as either feeding (1) or not feeding (0) by the CV pipeline. To establish the GT data, the feeding time records are converted into 98,720 cases with feeding (1) and not feeding (0) labels. The CV system achieved a precision of 87.22% and a recall of 89.09% with an 88.15% F1 score. Figure 3 presents a 40-min comparison between the CV predictions and the GT data. We randomly selected four (roughly 10-min each) time segments (7:41–7:51, 11:21–11:31, 15:30–15:40, and 19:32–19:42) for the visualization. The vertical axis of the figure represents the number of frames classified as feeding, while the horizontal axis indicates the time in minutes. For visualization purposes, the data is aggregated with 2-min intervals, hence where minutes without feeding events have a height of zero, and minutes with continuous feeding have a height of 1,200 frames. All subfigures demonstrate a close alignment between the CV predictions and the GT data under various lighting conditions, i.e. from morning to night. Some bunks have more visits than others at different times. However, it is important to note that the CV predictions exhibit some noise, primarily due to false-positive masks generated by the segmentation model.

**Figure 3:**
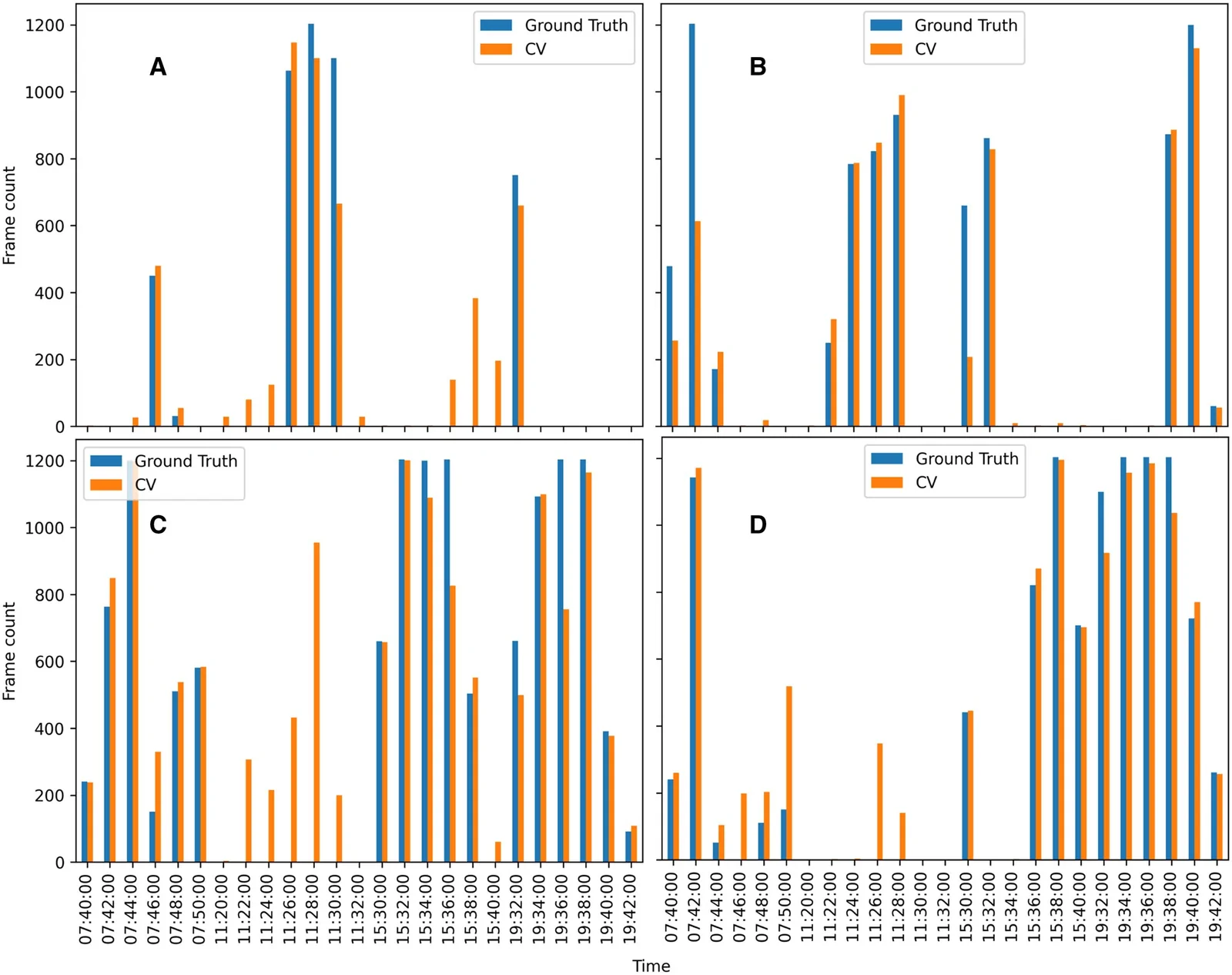
Comparison of CV and GT feeding at frame level. Each bar is aggregated by 2 min.

A limitation of this approach is that each frame is classified independently based on spatial overlap in that frame only, without incorporating temporal information, even though feeding is an inherently sequential behavior. This frame-independence likely contributes to the noise described above and may inflate or deflate performance. Incorporating temporal information, such as a sliding-window majority vote, could reduce this noise; however, selecting an appropriate window size and filtering strategy requires behavioral data on typical feeding about duration and frequency, which was not available in this study. We identify temporal modeling of frame-level predictions as a direction for future work.

### Case study 2: feed grading classification

Feed costs represent a significant portion (65–75%) of operational expenses in beef operations, making feed efficiency crucial for economic impact (Tedeschi et al. 2021). Thus, the scientific community must develop methods to monitor feed for reducing waste or improving profitability and sustainability. However, accurate scales are not widely applied in beef operations due to high cost and maintenance requirements (Wang et al. 2006). This study aims to automate the feed grading tasks using CV techniques in a data efficient way. Figure 4 shows the definition and visualization of the feed grades from S00 to S40. This definition is based on six bunk scores: S00 (no feed), S05 (scattered feed), S10 (thin layer), S20 (25–50%), S30 (>50%), and S40 (untouched) (Lundy et al. 2015).

**Figure 4:**
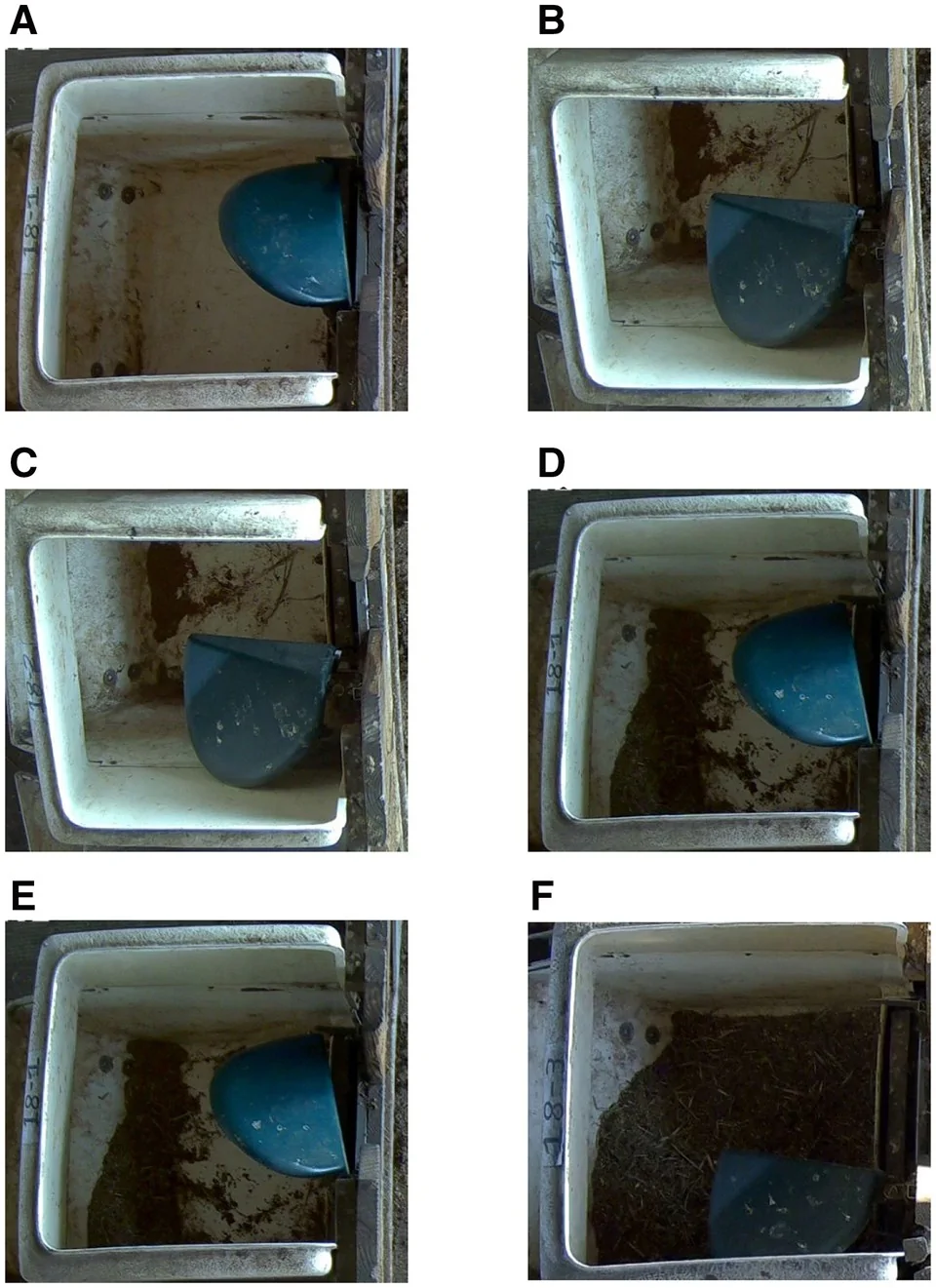
Feed grading annotation examples and description. A) S00: no feed remaining in the bunk, B) S05: scattered feed remaining. Most of the bottom of the bunk is exposed. C) S10: thin, uniform layer of feed remaining. About 1 com kernel deep. D) S20: 25–50% of feed remaining. E) S30: more than 50% of feed remains. The crown is thoroughly disturbed. F) S40: feed is virtually untouched. The crown of the feed is still noticeable.

The off-the-shelf approach in Case Study 1 used the existing class “cow” to detect the animals. However, domain-specific feed grade categories are not something an off-the-shelf model can classify without adaptation. Designing and training models from scratch costs lots of design time and labeled data. Instead, transfer learning can leverage the large CV datasets and well-designed models, to train a customized model with very little labeled data (Zhuang et al. 2021). Figure 5 shows the transfer learning process compared to training from scratch. The well-designed ResNet model is pre-trained on ImageNet dataset (Krizhevsky et al. 2012), comprising 1.3 million images spanning 1,000 classes. The ResNet weights and structure already encode generic visual features—edges, textures, and shapes—learned from this large-scale dataset. Transfer learning copies these weights and modifies the last output layer slightly to the new task using our dataset, requiring far less labeled data and training time than learning these features from scratch would demand. ResNet is one of the most successful classification model due to its residual block design to solve the degradation problem (He et al. 2016). For fast processing, the smallest ResNet ResNet18, i.e. with 18 layers, was selected with its weights pre-trained on ImageNet. The pretrained ResNet is available on most major AI platforms, such as TensorFlow and PyTorch with many different versions (ResNet50, ResNet 100, and so on). We performed full fine-tuning: all network weights were updated during training (not frozen), with only the final fully connected layer replaced to output the six feed-grade classes.

**Figure 5:**
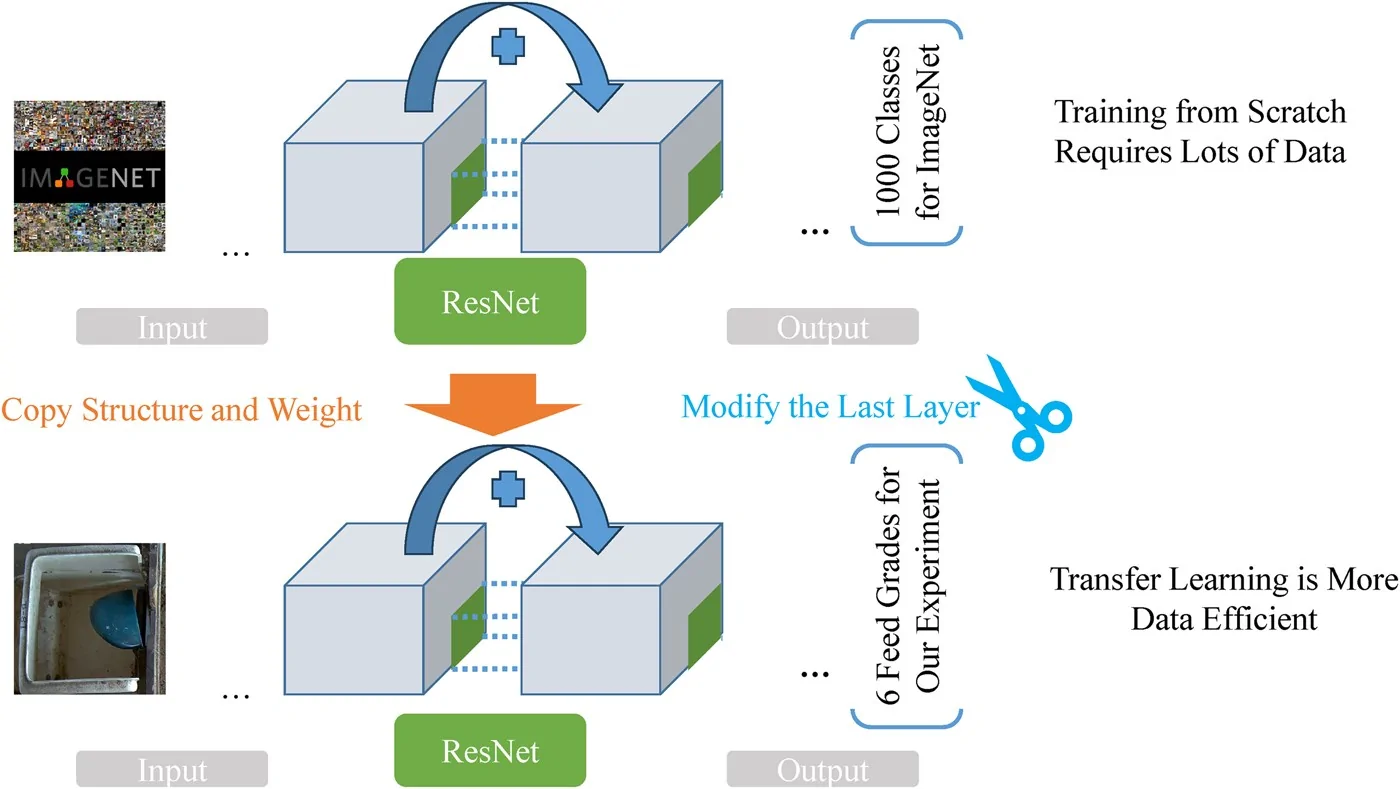
Comparison between transfer learning and training from scratch.

The dataset was collected in a research feedlot equipped with the Calan Feeding System, comprising six individual feeding bunks as shown in Figure 6A. To monitor the feeding behavior of the beef cattle, we installed a high-definition video capture system, which consisted of three Reolink PoE cameras (Model D400) positioned above the feeding bunks, with each camera capturing two bunks shown in Figure 6B. Consecutive frames were extracted from the videos and cropped to focus only on feeding bunks. The cameras were configured to record at a resolution of Full HD (1,920 × 1,080 pixels) with a frame rate of 30 FPS.

**Figure 6:**
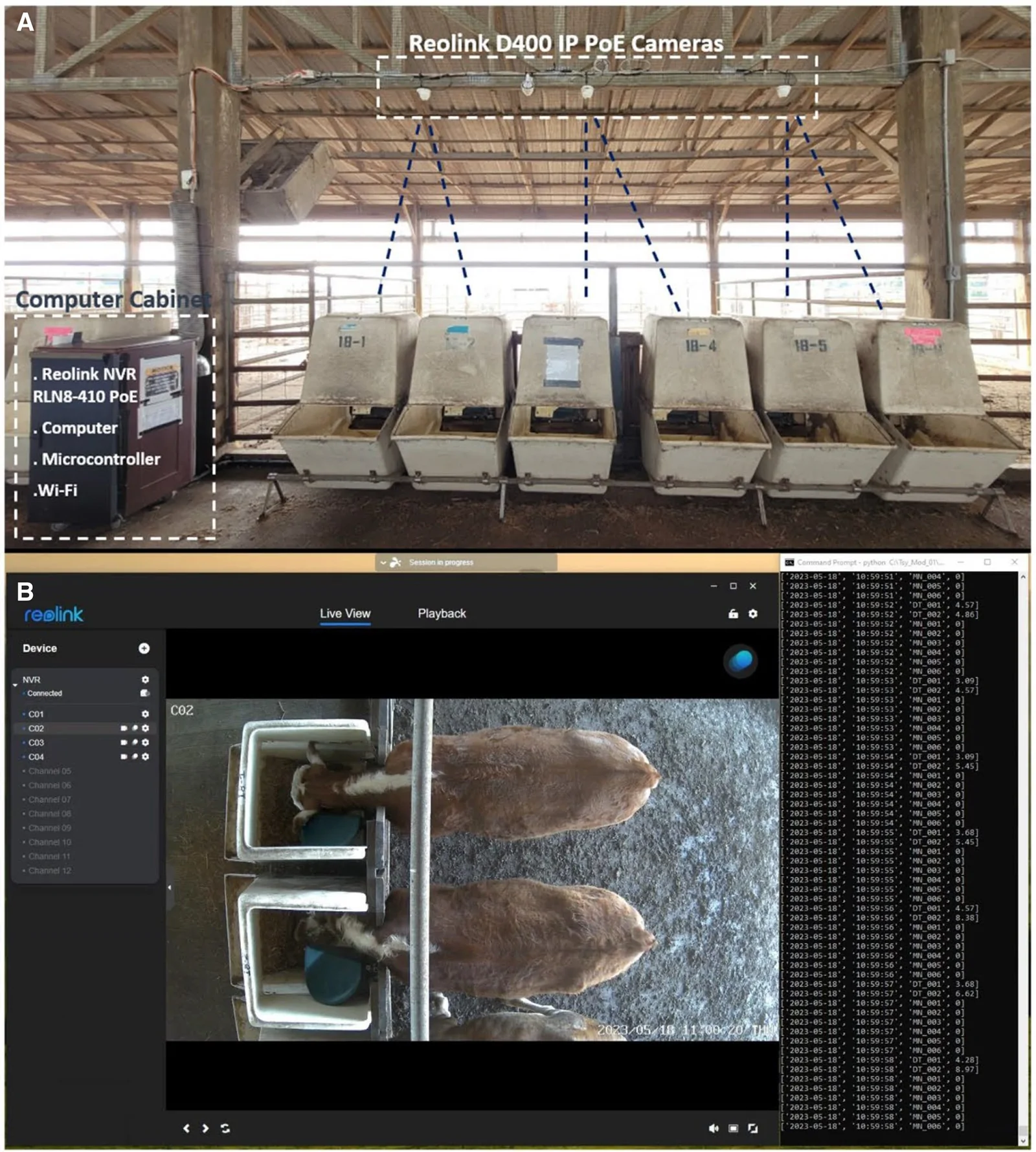
Panel A shows the Calan Feeding System with a video capture system positioned above the feeding bunks to capture animal activities. Panel B shows the top-down view of one camera.

By random sampling, we created a total dataset of 183 images for six classes labeled by the team’s animal science researcher spanning the three cameras and six feed bunks during different times of the day with varying light conditions. Table 1 lists the data distribution in the column training + validation + testing, and this small dataset is split into training (80%), validation (10%), and testing (10%). Importantly, this dataset is highly imbalanced: Class S40 has only seven samples. To balance, only the training images were augmented using a composed set of transformations, including random rotation between −15° and +15°, random affine transformation combining translation (up to 10% of image dimensions), scaling (0.9–1.1), shear (up to 10°), and random perspective distortion (distortion scale 0.2) applied with 50% probability. This resulted in the six classes all reaching 45 training samples, yielding a balanced augmented training set of 270 images. Additionally, we collected a diverse set of 7,905 frames (last column in Table 1) randomly sampled across different days and labeled them to enable an independent model performance verification. This extra labeled data is for performance verification only, not for model training and validation.

**Table 1:** Feed grading classification data size.

Class	Training + validation + testing	Original training	Augmented training	Verification
**S00**	18	13	45	906
**S05**	56	45	45	3,189
**S10**	39	24	45	1,338
**S20**	17	8	45	682
**S30**	46	33	45	1,253
**S40**	7	5	45	537

The model was trained on an NVIDIA A100 GPU on a High-Performance Computer (HPC) system. Training used the Adam optimizer with an initial learning rate of 0.0001, decayed by a factor of 0.1 every five epochs, batch size 4, and early stopping based on validation loss. The training required about 10 min, with early stopping to save the model with the best validation loss in 10 epochs. Besides data augmentation previously mentioned, the brightness of the frames was multiplied by a random factor between 0.5 and 1.5 to mimic the light change.

As expected, ResNet with transfer learning stopped training at around 88% validation and testing accuracy. When tested more extensively, the model achieved an overall accuracy of 91% on the verification dataset (7,905 frames), as shown in the confusion matrix in Figure 7. In this matrix, each column is the labeled class, and each row indicates the predicted class. Table 2 shows the class-wise precision, recall, and F1 scores: Classes S00 and S05 performed very good with 97–100% F1 scores. A small portion of S10 was wrongly predicted as S05. Class S00 had the best prediction because of the clear appearance of an empty bunk. Class S40 and S30 are the weakest overall, driven by the similar appearance, which is also difficult for human definition. The macro average (F1 = 89.5%) treats all classes equally, while the weighted average (F1 = 91.41%) is influenced by the two best-performing and most frequent classes, S00, and S05. Altogether, the model established on 183 labeled images showed reliable performance on the verification data set making it data efficient. In contrast, confusion among S20, S30, and S40 was more common because the distinction between these classes can be subjective, especially when feed distribution is uneven. It should be noted that the proposed method is data efficient but not entirely data independent. The approach relies on pretrained ResNet weights obtained from ImageNet. Without pretrained weights, substantially more task-specific labeled data would be required to achieve comparable performance.

**Figure 7:**
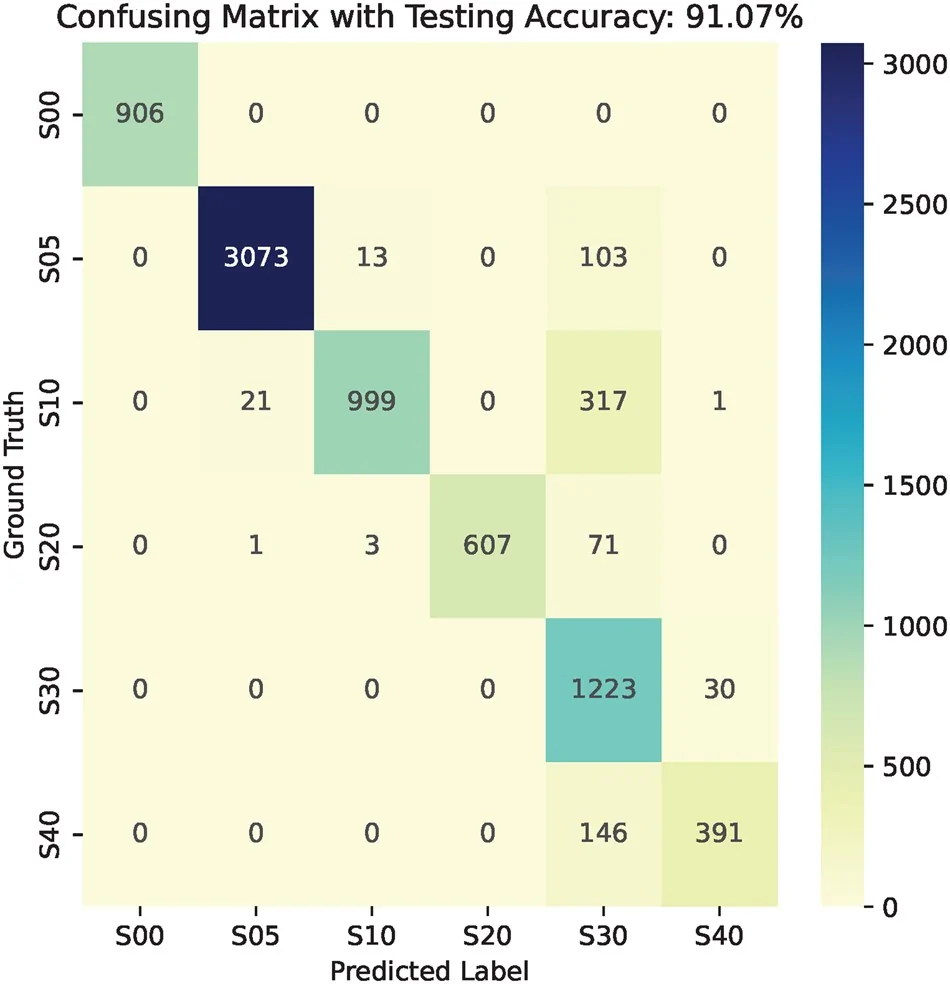
Feed grading confusion matrix.

**Table 2:** Feed grade verification results: precision, recall, and F1 scores, macro average, and weighted average.

Class	Precision	Recall	F1-score
**S00**	100.00%	100.00%	100.00%
**S05**	99.29%	96.36%	97.80%
**S10**	98.42%	74.66%	84.91%
**S20**	100.00%	89.00%	94.18%
**S30**	65.75%	97.61%	78.57%
**S40**	92.65%	72.81%	81.54%
**Macro average**	92.69%	88.41%	89.50%
**Weighted average**	93.52%	91.07%	91.41%

### Case study 3: individual animal identification

Animal identification is an indispensable part of PLF management (Paudel and Brown-Brandl 2025). Radio frequency identification (RFID) sensors are needed to track individual animals, which requires frequent installation and maintenance due to incoming and outgoing animal production (Halachmi et al. 2019). Some animal identification research has shown the potential of visual signals in animal identification (Bergman et al. 2024; Bumbálek et al. 2025). However, most of them rely on manual labeling each animal as a class. Given the difficulty of labeling all animals in practice, this study tries CV to identify individual animals without labeling. Figure 8 shows the CV feed-sensing design that combines feed grading from the previous study and label-free animal identification. For animal frames, this work proposes using a statistical method to cluster similar frames into separate groups corresponding to different animals, based on features extracted. Feature extraction here means running each animal frame through a pre-trained ResNet (typically ImageNet-trained) and taking second-to-last layer that captures the image’s visual appearance without using the network’s classification output.

**Figure 8:**
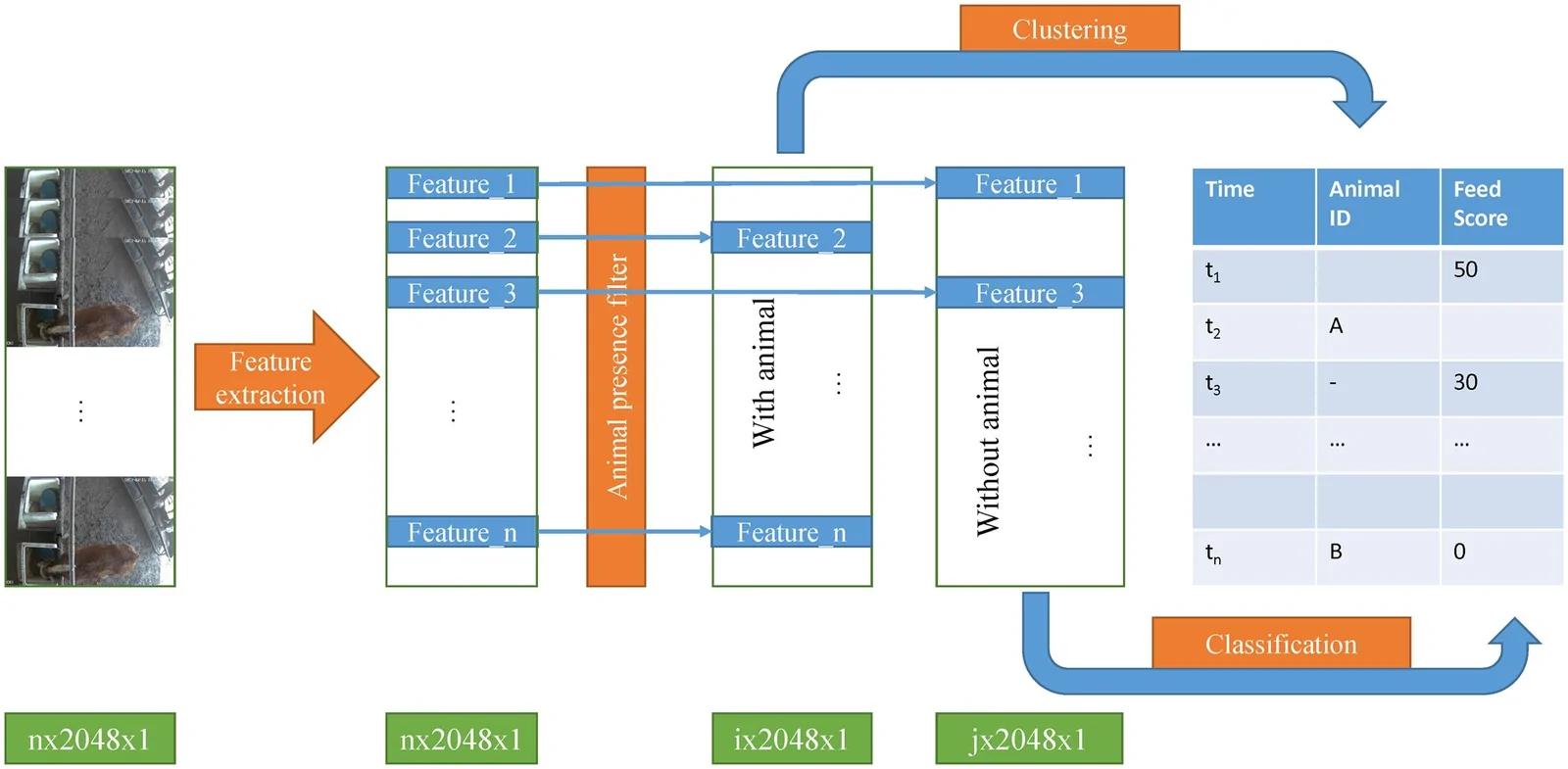
Feed sensing design.

There are two application cases to tell animals apart: when the total number of animals is known or unknown, leading to two different methods for clustering the animals. The goal is to evaluate whether visual features extracted contain sufficient animal-specific information to support label-free identification. Such frame-level clustering could aid long-term re-identification because many object tracking algorithms, such as Deep-SORT suffer from ID switches over long times (Wojke et al. 2017). When the total animal number is known, k-means clustering is a good way to group different animal images, as shown in equation 1. Here, m is the number of different animals, n represents the frame number, $u_{i}$ indicate the center of each cluster $c_{i}$, and *f_j_* is the feature of each image j. It is worth noting that $u_{i}\in R^{d}$ this means any feature dimension would work.


(1) $$c^{*}=\underset{c}{\mathrm{argmin}}\sum_{i=1}^{m}\sum_{f_{j}∊c_{i}}^{n}∥f_{j}-u_{i}{∥}^{2}$$

When the total number of animals is unknown, density-based spatial clustering of applications with noise (DBSCAN) is used to discover clusters as expressed in equation 2. Here, EPS(p) is the core point of one feature group corresponding to one individual animal. The features$\;f_{p}$ and$\;f_{q}$ are extracted from any frame p and q, σ is any distance metric, and ϵ is a user-defined radius. DBSCAN automatically determines how many animals can be clustered, and data points outside the radius will be considered as outliers and ignored. Meanwhile, the clustering result also needs to satisfy equation 3, where minPts is another user-defined parameter to constrain the minimum size of each group.


(2) $$\mathrm{EPS}(p)=\{q|\sigma (f_{p},\;f_{q})\leq \epsilon )\}$$

(3) EPS(q)≥minPts

The feed grading task was done by applying the ResNet model previously mentioned. The feed score difference between different times could be indicating feed consumption by the present animals. Altogether, the information will be stored in the table shown in Figure 8, which has time$\;t_{n}$, animal IDs, and feed grade scores. These data can be used to monitor individual animal bunk visits, feed intake, etc. Together, our design takes three steps: Animal presence sensing, feed grading classification, and animal ID clustering. The feature extraction was the same for all three steps, and the resulting feature $f_{n}\in R^{2048}$ based on the ResNet18 model pre-trained on ImageNet. The video data used were recorded on August 22, 2023, from 10:37:23 to 11:27:21. During this period, three animals, named AN-02, AN-05, and AN-06, visited one bunk (Feed Bunk #3), and the videos recorded from top-down. The GT data, including feed grade, animal ID, and animal presence, were generated by sensors and verified by the animal science researcher on the team.

#### Animal presence sensing

There was no need to use all the frames with 30 FPS because they were consecutive and highly similar images. Thus, we selected every 120th frame to use, because at lower frequencies (e.g. every second or every 30 frames), many of the images were nearly identical, and there was more variation at higher frequencies. This resulted in a dataset of 525 images, i.e. a 525 × 2,048 array for animal presence sensing. It was possible to deduce the presence of an animal using methods such as k-means, given that there were only two clusters: animal present and absent.

We used t-distributed stochastic neighbor embedding (t-SNE) (Van der Maaten and Hinton 2008) for dimension reduction, which resulted in a smaller array of size 525 × 2. This means that each of the 525 images now has a feature in two dimensions. Plotting one dimension along the horizontal axis and the other on the vertical axis showed three groups in Figure 9. This indicates we could automatically cluster the frames into three classes: empty bunks (no animal), full bunks (no animal), and with animals present. We also tried principal component analysis (PCA) (Martinez and Kak 2001) with k-means with k=2, which resulted in an adjusted rand index (ARI) of 0.99 when benchmarked with human separation. The shortcoming of such methods was that we still needed to manually inspect each cluster to determine which group had the animal and which did not.

**Figure 9:**
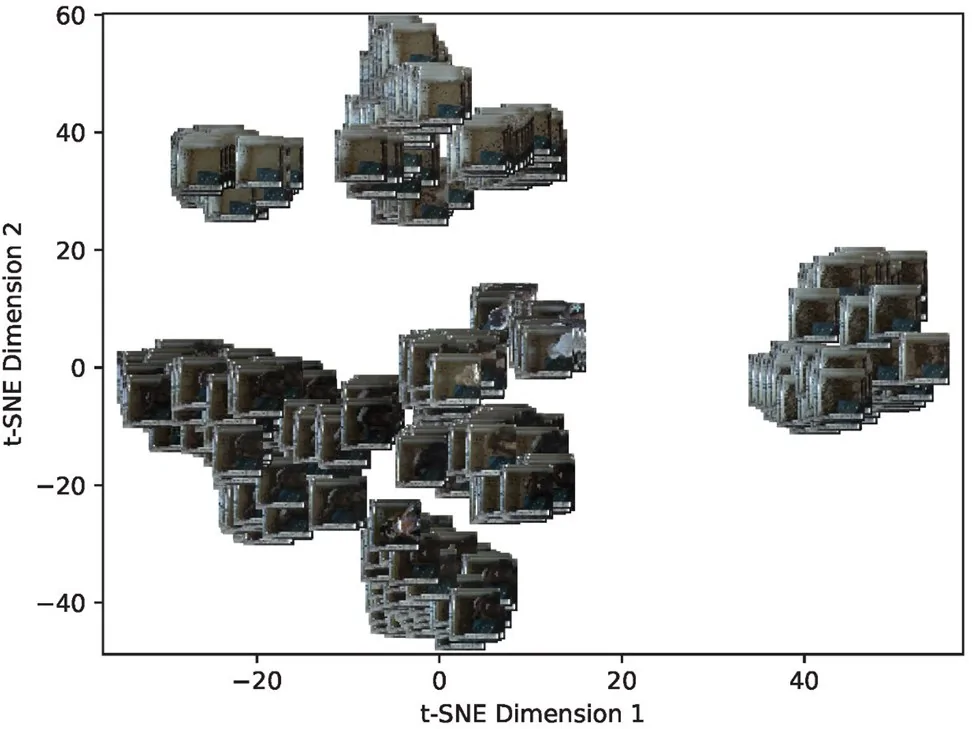
Visualization with t-SNE for all the frames.

To fully automate, we chose a simple supervised ResNet18 for animal presence sensing to maintain the focus of this study to be testing animal identification performance. Like the previous procedures, we used 2,400 frames (886 without an animal and 1,514 with an animal) collected outside the time window for training, validation, and testing. Due to the simplicity of the task, we only trained it for four epochs, with a learning rate of 0.001, momentum of 0.9, and standard multiclass cross-entropy for the loss function. Table 3 shows the confusion matrix of this ResNet tested on our 525 frames. This indicates robust performance (nearly 100% accuracy) when sensing the animal’s presence.

**Table 3:** Animal present classification confusion matrix.

Method	Present	Absent
**Present**	302	1
**Absent**	0	177

#### Animal ID

The animal presence sensing ResNet predicted 302 frames out of the total 525 containing animals. The images corresponding to the “no animal” class were directed to the feed grading ResNet previously described. For animal identification, we used k-means clustering with the number of clusters set to four (for the known animal number of three plus another missing value in case the ResNet was not perfect). For both k-means and DBSCAN methods, we tried three inputs: t-SNE linearly transformed into two dimensions, PCA reduced two dimensions, and the raw 2048-dimensional features. Table 4 shows the ARI scores for these two approaches. Compared to GT, the t-SNE features with DBSCAN achieved the best ARI of 0.96, indicating successful individual animal identification. K-means clustering yielded its best ARI of 0.87 with raw features. Figure 10 shows the DBSCAN clustering visualization with three different animals, with failure (first row) and success cases (second and third rows): While animals AN-02 and AN-05 were successfully separated, some AN-05 were identified as AN-06. Moreover, the success of DBSCAN was promising because it did not rely on the known animal numbers unlike k-means.

**Figure 10:**
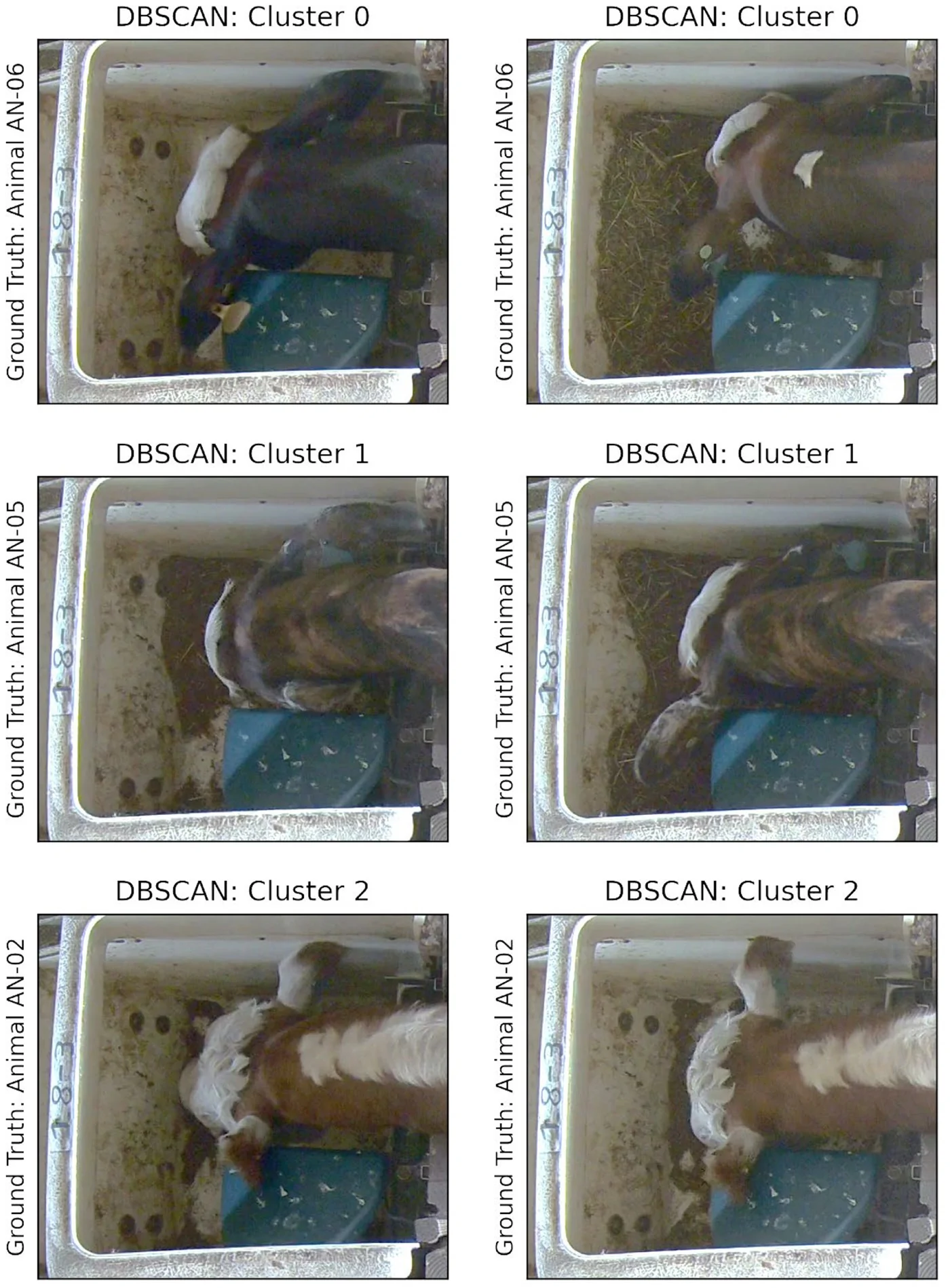
Animal ID examples. The animals that appear in the testing dataset are AN-06, AN-05, and AN-02 at the same bunk.

**Table 4:** Adjusted Rand Index (ARI) scores for animal clustering.

Method	t-SNE	PCA	Raw
**DB-SCAN**	0.96	0.85	0.83
**K-means**	0.78	0.69	0.87

Two-dimension reduction techniques, i.e. t-distributed stochastic neighbor embedding (t-SNE) and principal component analysis (PCA) are used.

#### Final results

Figure 11 shows the feed grading score on the vertical axis with time on the horizontal axis as the result visualization. The individual animal IDs were color-coded on the negative side of the vertical axis. Notice that the frames without animals were also coded as feed grade for visualization. This average processing time per frame of the AI model is 0.0003 s on a NIVIDIA T4 GPU, translating to 3,000 FPS (100 times faster than normal 30 FPS), making it real-time information retrieval methods for farm management. Over the video duration, the CV framework identified the feeding grade as S30 at the beginning, with little noisy prediction to be S40, decreased to S10 in the middle, and reached S05 at the end. This visualization was aggregated with a 4-s interval for better visualization. In between, three animals appeared at the bunk. If feed-grade changes were assumed to be attributable to animals present at the bunk, an assumption this research does not currently validate, animal feeding intake could potentially be estimated from this graph.

**Figure 11:**
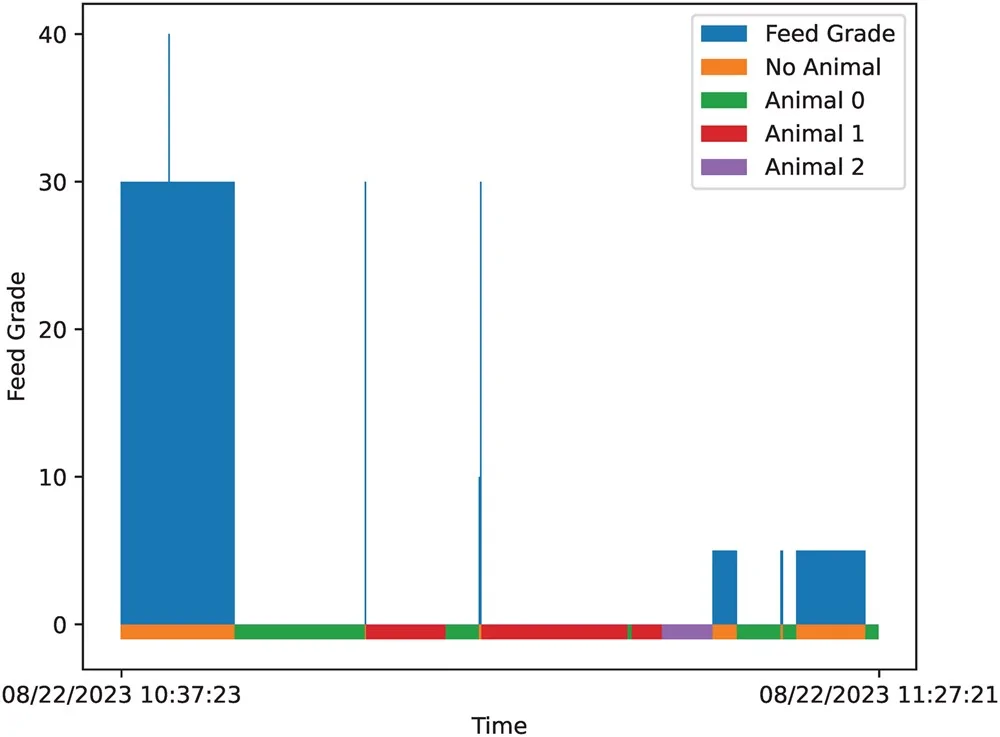
Feed grading score (S00–S40, vertical axis) predicted by the computer vision framework over a representative 1-h sample video (horizontal axis), aggregated into 4-s intervals. Blue bars show the aggregated feed grade and illustrate frame-to-frame prediction noise. The colored band along the zero line indicates bunk occupancy during each interval: orange = no animal present; green, red, and purple = individual animal IDs 0, 1, and 2, respectively.

## Current challenges, future directions, and research needs

### Individual animal identification

Many recent PLF pilot studies in academia and industry have demonstrated the feasibility of using CV to monitor animal activity, key points, body weight, and related parameters (Bergman et al. 2024; Menezes et al. 2024). All these tasks are based on short-term individual animal identification, where each animal is treated as a class in the visual dataset. However, such methods fall short in practice due to long-duration induced ID switches, changes in animal appearance over time, and variations in video visual signals, including weather, camera resolution, angle, and pen configurations. Moreover, livestock are subjected to continuous growth and turnover cycles driven by production demands, involving entry and exit from the agricultural system and requiring new labels for new animals, further challenging current CV models. Furthermore, there is a lack of an open-source, large-scale, and highly diverse dataset that enables individual animal identification and tracking research. Such datasets will serve as a testing bed as well as validation for CV innovations. PLF management such as poultry, dairy, and equine monitoring can also benefit from the investigation of such large-scale datasets.

### Model robustness, generalizability, and interpretability

Although high internal classification accuracies were achieved in the presented case studies, deep learning models are susceptible to domain shift when deployed across farms with differing lighting conditions, camera configurations, breeds, coat patterns, stocking density, and environmental variability. Cross-site validation and seasonal robustness testing are essential to ensure model transferability beyond the training environment. Moreover, performance metrics alone do not guarantee responsible deployment. DNNs are often criticized for their limited interpretability, particularly in high-stakes decision contexts (Rudin 2019). The integration of explainable AI frameworks, such as feature attribution methods (Lundberg and Lee 2017), can improve transparency and producer trust. Broader discussions on robustness, generalizability, and interpretability challenges in livestock AI systems further emphasize the need for biologically grounded and externally validated approaches (Condotta and Tedeschi 2026).

### Multi-spectrum imaging

Most current CV studies in PLF rely on cameras for visible red, green, and blue (RGB) light (roughly, wavelengths from 370 to 730 nanometers). The infrared spectrum has a broader range which is temperature sensitive. From 0.73 to 3 µm, the near infrared (NIR) and short-wave infrared (SWIR) bands are close to the visible spectrum, and the light is mostly reflected in radiation. Light with wavelengths from 3 to 15 µm, mid-wave infrared (MWIR) and long-wave infrared (LWIR) are mainly self-emitted heat (Teutsch et al. 2021). It is known that including infrared signals improves CV performance in plants (Niu and Chen 2023) and animals (Bresolin and Dórea 2020). Considering the unique temperature pattern of animals, it is possible to combine MWIR and LWIR signals with RGB to improve the individual animal identification and perhaps disease detection.

### The vision data challenge in PLF

Generating labeled datasets for long-duration livestock monitoring is a fundamental bottleneck in PLF research. Manual annotation is time-intensive and does not scale, and individual animal identification is particularly demanding: Prior attempts by the authors confirmed that frame-by-frame labeling of individual animals across production cycles is impractical. Yet mathematical modeling and machine learning both depend on high-quality, large-scale data, making dataset creation a prerequisite for progress. Several technical approaches show promises for reducing this burden. Depth estimation cameras can measure the relative spatial positions of animals within a pen, providing geometric cues that complement visual appearance. Key point detection can characterize individual gait patterns, potentially serving as a biometric signature. Barcodes or other machine-readable markers physically placed on animals could streamline identity labeling at scale, while radio-based technologies such as RFID and received signal strength indicator (RSSI) offer a path to automatically generating large volumes of paired animal-identity data without manual video review.

Beyond visual signals, multimodal data fusion offers additional opportunities. Multimodal approaches such as contrastive language-image pre-training (CLIP) (Radford et al. 2021) fuse data formats such as text, images, and video in the same space (Vysyaraju et al. 2025). More recently, work on animal vocalization has demonstrated that audio signals carry information relevant to animal welfare (Lekhak et al. 2025; Wang et al. 2025), suggesting that audio-language processing could supplement camera-based monitoring with an independent sensing channel. Integrating visual, thermal, audio, and text data into a unified framework represents a natural direction for the field and could transform individual animal identification from a CV problem into a richer, multi-signal inference task.

## Discussion

We demonstrated that CV techniques can sense animal presence, grade feed levels, and identify individual animals at the bunk from standard camera footage alone with minimum manual label effort. Case study 1 showed the off-the-shelf CV models MASK-RCNN can generalize to other unseen PLF settings and extract useful information with 88.2% F1 score compared to physical bunk sensor GT. By using transfer learning, Case Study 2 showed robust performance can be achieved with limited data annotation effort (a few hundred labels). Feed-grade classification across six bunk score levels achieved 91% accuracy on an independently collected verification dataset, with performance strongest for unambiguous grades, such as empty and full bunks. Case study 3 showed statistical clustering could be integrated together with feed grading to produce individual animal feed data. DBSCAN and k-means clustering of ResNet-extracted features achieved an ARI up to 0.96 against expert-verified GT, successfully differentiating animals without any animal-level annotation. Taken together, these results showed that meaningful individual-level livestock data can be extracted using only cameras and limited data annotation.

However, the reviewed case studies were conducted on a relatively small scale with several animals monitored over a few days. Given the encouraging results, the natural next step is to expand the methods to a pool of diverse animal and farm conditions. Individual animal identification remains the foundational capability on which downstream PLF monitoring tasks all depend on, and the results here suggest that data-efficient, camera-based approaches are a viable path toward achieving it. Future work should extend the framework to wider pen views with multi-camera tracking, incorporate thermal and audio sensing channels to improve robustness and support disease detection, and develop large-scale open datasets to accelerate the research progress across the field.

## Supplementary Material

skag316_Supplementary_Data

## Data Availability

The dataset and accompanying Python code are openly accessible at: https://github.com/TAMIDSpiyalong/2025ASAS/blob/main/computer_vision_for_feed_grading.ipynb.

## Abbreviations

AI: artificial intelligence
ARI: adjusted rand index
CLIP: contrastive language-image pre-training
CNN: convolutional neural network
COCO: common objects in context
CV: computer vision
DBSCAN: density-based spatial clustering of applications with noise
FN: false negative
FP: false positive
FPS: frames per second
GAN: generative adversarial network
GPU: graphics computing unit
GT: ground truth
Mask RCNN: mask region-based convolutional neural network
NN: neural network
PCA: principal component analysis
PLF: precision livestock farming
RFID: radio frequency identification
RGB: red, green, blue
RSSI: received signal strength indicator
SSD: signal shot detection
TN: true negative
TP: true positive
T-SNE: t-distributed stochastic neighbor embedding
YOLO: you only look once
