# Supplementary material for: ASAS-NANP SYMPOSIUM: mathematical modeling in animal nutrition—data-efficient computer vision for precision livestock farming
Source: J Anim Sci. 2026 Sep 29;104:skag316. doi: 10.1093/jas/skag316 (PMC13700060; doi:10.1093/jas/skag316)
Supplement: Supplementary file 1 [file skag316_supplementary_data.zip › 20260813 Appendix.docx]

**APPENDIX 1: ARTIFICIAL INTELLIGENCE AND COMPUTER VISION FUNDAMENTALS**

***Neural Network Fundamentals***

This appendix reviews AI fundamentals with a focus on CV technologies and discusses their potential functionalities for practical implementation. Advances in AI, and its applications in general, including self-driving cars, large language models, and recommendation systems, are all based on NN to model the high-dimensional mathematical relationships between digital inputs and outputs. Because neural networks are highly nonlinear models, the resulting optimization problem is generally nonconvex and does not have a closed-form analytical solution. Instead, parameters are iteratively updated using optimization algorithms such as gradient descent and its variants. In addition to model parameters, network performance is influenced by hyperparameters, including learning rate, network architecture, batch size, and regularization settings, which must be selected prior to training and tuned to achieve optimal performance.

Figure A1 illustrates a generic neural network architecture that maps an input vector $\vec{x}$ to the output vector $\vec{y}$. Both $\vec{x}$ and $\vec{y}$ may be multidimensional vectors whose dimensions depend on the specific task. For example, $\vec{y}$ may represent a single prediction value, a set of class probabilities, or multiple predicted variables. This makes NN applicable to a wide range of problems. Each circle $H_{k}$, all yellow circles, in between the input and output layer is one number holder, a.k.a. neuron in literature. The weights $\vec{w}$, another set of numbers represented by solid lines connecting neurons, is used to calculate each $H_{k}$. They are named $w$ because $H_{k}$ is the weighted sum of the previous neurons. Equation A1 shows how one neuron $H_{k}$ is computed where $i$ indicate the input neuron from the previous layer, $j$ represents individual weight from previous layer to $H_{k}$, and $b$ is the bias term. This is the same for all other neurons running from first to $k$-th. It is obvious that the successful $\vec{y}$ depends on the right collection of $\vec{w}$.

$$H_{k}=\sigma(x_{1}*w_{1}+x_{2}*w_{2}+\ldots+x_{i}*w_{j}+b)$$

(A1)

To optimize the model, a mathematical technique called gradient descent, shown in Equation (A2), is commonly used to train neural networks by iteratively updating model parameters to minimize the loss function. The process of finding the optimum weights is often called training with known pairs of output $\vec{y}$ given input $\vec{x}$. Starting from randomized weights, the prediction $\vec{y_{pred}}$ will be compared with the ground truth $\vec{y}$ using a loss function. For example, mean square error $MSE =\frac{1}{n}\sum_{i=1}^{n} \left( \vec{y_{pred_{i}}}-\vec{y}_{i} \right)^{2}$for each pair $i$ is normally used in simple regression problems. To make the network nonlinear, the activation function $\sigma$ in Equation (A1) transforms the weighted input before passing it to the next layer. Common activation functions include ReLU, sigmoid, tanh, and Leaky ReLU, each introducing nonlinearity in a different way. This nonlinear transformation enables neural networks to learn complex relationships in data and effectively model nonlinear problems.

Formally, the change of loss with respect to each weight is called the partial derivative, written as  $\vec{\frac{\partial loss}{\partial w_{ij}}}$, where $i$ and $j$ index each weight (solid lines). It helps to think of this as a collection of small changes $\nabla$, which have the same dimension as $\vec{w}$, so each weight makes a small change to minimize the total loss. This collection, multiplied by a very small factor called the learning rate (LR), e.g., 0.001, is used to update the  $\vec{w}$ in many iterations until the loss plateaus. When the neural network is huge with millions of $w$, parallel computing becomes important for efficiency, as the hardware requirements for GPUs nowadays.

$$\vec{w (new)}=\vec{w (old)}-\nabla*LR$$

(A2)

***Classification Problems and Metrics***

Classification tasks are important in many AI applications due to their simplicity. For example, NLP models predict the most likely next word given the previous words. The NLP prediction is a list of probabilities corresponding to the entire vocabulary, with the highest probability corresponding to the most likely next word. This predicted probability list is typically normalized so that its sum is 1 (100%), representing mutually exclusive classes. Correspondingly, the ground truth output or labels are arranged so that a positive case is represented by 1 and a negative case by 0, known as one-hot encoding.

A common way to measure classification loss is the summation of the negative log‑likelihoods for all predictions: $loss=-$ $\sum_{1}^{i} y_{i}log(p_{i})$ , where $p_{i}$ is the prediction and $y_{i}$ is the ground truth label for each prediction case $i$. During model training, the optimization process seeks to minimize this loss, which encourages higher predicted probabilities for the correct class and lower probabilities for incorrect classes.

Regression model performance can be evaluated using several metrics, including mean squared error (MSE), root mean squared error (RMSE), mean absolute error (MAE), and the coefficient of determination (R²). Some of these metrics, such as MSE, are also commonly used as loss functions during model training, but evaluation metrics and loss functions serve different purposes.

Classification models require separate evaluation metrics that quantify predictive performance. For binary classification, predictions are summarized using a confusion matrix consisting of four outcomes: true positive (TP), false positive (FP), true negative (TN), and false negative (FN). Based on these counts, metrics such as accuracy, precision, recall, and F1-score can be computed. For multiclass classification, these metrics are typically calculated for each class and then aggregated using methods such as macro, micro, or weighted averaging. Equations (A3-6) present the definitions of accuracy, precision, recall, and F1-score, which are among the most reported metrics for classification tasks.

$$accuracy=\frac{TP+TN}{TP+FP+TN+FN}$$

(A3)

$$precision=\frac{TP}{TP+FP}$$

(A4)

$$recall=\frac{TP}{TP+FN}$$

(A5)

$$F1 =2*\frac{precision*recall}{precision+recall}$$

(A6)

***Principles of Computer Vision***

Figure A2 shows the general architecture of a convolutional neural network (CNN) model. The input is a $w\times h\times c$ matrix with the width, height, and channel dimensions. The standard red, green, and blue (RGB) image has a channel $c=3,$ and $w\times h$ pixel size. Each pixel has a value ranging from 0 (darkest) to 255 (brightest) to represent the light intensity reflected by objects. With many trainable weights $\vec{w}$ in the convolutional (CONV) kernels. Next, a max pooling (MAX) operation is applied to shrink the image by a factor of 2 or more by selecting the highest values in a grid (e.g., 2x2 pixels). There is no trainable weight in this step. After a series of combinations of CONV and MAX, the final output is a list of probabilities (normalized to 0 and 100%) corresponding to the target class $n$ for classification tasks. The training process still follows Equation A2 with millions of images paired. CNN models are more complicated than plain DNN models, however the weight training, deployment, and validation are based on the same principle.
